# Cognitive and motor disturbances in depression: insights from comprehensive behavioral assessments

**DOI:** 10.3389/fpsyt.2025.1624776

**Published:** 2025-07-29

**Authors:** Ioanna Douka, Marit F. L. Ruitenberg, Kamile Weischedel, Carlos Phouthavongsay, Sara L. Weisenbach, Jos N. van der Geest, Brian J. Mickey, Vincent Koppelmans

**Affiliations:** ^1^ Department of Psychiatry, Huntsman Mental Health Institute, University of Utah, Salt Lake City, UT, United States; ^2^ Department of Health, Medical and Neuropsychology, Leiden University, Leiden, Netherlands; ^3^ Leiden Institute for Brain and Cognition, Leiden, Netherlands; ^4^ McLean Hospital, Harvard University, Belmont, MA, United States; ^5^ Department of Neuroscience, Erasmus MC, Rotterdam, Netherlands

**Keywords:** cognition, depression, motor functioning, psychomotor disturbances, fine motor

## Abstract

**Background:**

Depression affects not only mood and reward processing, but also motor and cognitive functioning, leading to psychomotor disturbances crucial for diagnosis, prognosis, and treatment. Patients with severe psychomotor retardation often respond poorly to SSRIs but benefit from neurostimulation like ECT. However, comprehensive assessments of cognitive and motor domains in the same depression sample are rare.

**Methods:**

This pilot study compared 20 depressed patients and 22 controls across multiple tests of cognitive and motor functions. We examined executive function and processing speed (Symbol Digit Modalities Test (SDMT), D-KEFS Color-Word Interference Test), verbal and visual learning and memory (Hopkins Verbal Learning Test (HVLT-R), and Brief Visuospatial Test (BVMT-R), gait (2-minute walking, 4-meter walking and walking while talking (WWT) tests), sarcopenia (grip strength ftest, knee extension test), and fine motor function (Archimedes Spiral Test, 9 Hole Peg Test). Associations between depression severity and behavioral performance were also explored.

**Results:**

Depressed participants performed significantly worse on the color naming and interference conditions within the D-KEFS Color-Word Interference Test and on the HVLT's delayed recall. They were slower on the 9 Hole Peg Test with both their dominant and nondominant hands, while no differences were noted in gait or sarcopenia. Greater depression severity correlated with poorer performance on the WWT dual cognitive-motor task and quicker movement on the Archimedes Spiral task.

**Conclusions:**

These findings reveal decrements in cognitive and motor domains in depressed individuals, which could impact daily functioning. Overall, results from this pilot study suggest that examining motor disturbances alongside cognitive disturbances could serve as a marker of disease progression and a potential target for intervention.

## 1 Introduction

A depressive episode is defined by low mood or anhedonia (i.e., loss of interest in normally enjoyable activities), and can be accompanied by decreased energy, concentration problems, psychomotor retardation or agitation, changes in appetite or sleep, increased feelings of worthlessness, guilt, or thoughts of death and suicide (1). For a clinical diagnosis, the constellation of the above symptoms has to be linked to substantial functional impairment and be present for at least two weeks (2). Functional impairment as a result of depression is apparent in daily living activities, interpersonal relationships, work, and scholarly productivity (3, 4). Notably, depression is one of the leading causes of disability worldwide (5, 6). The economic burden of US adults with depression was calculated to be $326 billion in 2018 (7). The high prevalence of this severely disabling disease in combination with the large economic impact warrants the development of novel treatments. In an effort to improve the understanding and treatment of mood disorders, the National Institute of Mental Health developed Research Domain Criteria (RDoC), aiming to identify dimensional models of psychopathology instead of using hard symptom cutoffs for the diagnosis of mood disorders (8, 9). More recently, psychomotor abnormalities were added as a novel RDoC domain (10), highlighting the value of studying psychomotor disturbances as predictor and outcome measures in depression.

Psychomotor abnormalities are known to present in depression as psychomotor retardation or agitation, and their presence typically indicates a more severe disease phenotype (11). Psychomotor retardation in depression can present as slower speed of movement, reduced speaking rate, lack of motor initiation, body immobility, postural abnormalities, loss of facial expressions, and cognitive ideation (12). Psychomotor agitation can present as increased activity, motor restlessness or irritability, characterized by aimless, non-purposeful movements (13, 14). These impairments reflect both a neuromotor and a higher-level cognitive component. Motor deficits that have been observed in depression that can affect both treatment outcomes and daily functioning, independent of motivational factors, include gait impairments, sarcopenia (e.g., grip strength), and poorer fine motor skills (15). Although the motor component of psychomotor disturbances has been recognized as a potential fundamental dimension of depression, prior studies lack comprehensive assessments across all motor domains and, to our knowledge, objective and quantifiable motor profiles are not currently being developed for depression. Motor deficits in depression may, to a certain degree, reflect impairments in cognitive functioning. It has already been established that successful motor performance does not only require the direct, physical control of muscles, but also involves cognitive processes that allow us to engage in goal-directed behavior (16, 17). Especially higher order psychomotor functions that depend on intact cognition, such as visuospatial function, attention, working memory, and executive functioning (18). Dual-task studies show that increased cognitive load worsens motor performance, supporting this interaction (19, 20). Motor and cognitive functions are closely linked via shared neural circuits, and disruptions in one can impact the other, such as driving a car (21, 22). Cognitive impairment also plays a key role in the overall functional impairment associated with depression. More importantly, cognitive dysfunction and thus functional impairment to some degree persist even after improvement of depressive symptoms (23, 24). This is independent from disease severity and can be long-lasting (3). Additionally, cognitive dysfunction not only prevents a full recovery from depression, but it also increases the risk for recurrence of episodes (25, 26).

Over the years, several subtypes of depression have been proposed and identified for patients exhibiting more significant psychomotor impairment (e.g., atypical, melancholic depression) (27). Patients presenting with these psychomotor-related phenotypes respond better to specific antidepressant pharmacotherapy (e.g. tricyclic antidepressants (TCAs), monoamine oxidase inhibitors (MAOIs)) (28), which are different from what is usually used as a first line treatment in a major depressive episode (i.e., SSRIs) (29). Also, evidence suggests that patients with this depressed phenotype respond better to neurostimulation interventions (e.g., electroconvulsive therapy) (30). Moreover, studies support that the presence of severe psychomotor impairment in a depressive episode might indicate the presence of bipolar disorder rather than major depressive disorder, suggesting potential utility for differential diagnosis (31). Overall better characterization and understanding of psychomotor abnormalities has great diagnostic, prognostic, and treatment importance for depression.

To identify which cognitive and motor functions are most strongly affected by depression, performance across the breadth of domains should be evaluated. The present pilot study explores the impact of depression on cognitive and motor functions by comparing performance between adults with a current depressive episodes and control subjects across a series of objective cognitive and motor tasks, including those evaluating executive function, processing speed, verbal and visual learning and memory, gait, sarcopenia, and fine motor function. These domains, rarely examined together within a single sample of people with depression, could reveal a pattern of subtle cognitive and motor deficits, which could directly be linked to daily functioning. Relative to clinician-rated tools, our approach offers ecologically more valid measures to capture such a broad motor profile in this population, potentially supporting earlier detection and intervention.

We hypothesized that depressed individuals would perform worse on a series of cognitive tasks as well as a variety of motor measures than controls, specifically on tasks that measure motor speed. We further examined the association between depression symptom severity and behavioral performance within the depressed sample, hypothesizing that more severe depression is related to poorer psychomotor performance.

## 2 Methods

### 2.1 Participants

A total of 20 individuals with a major depressive episode and 22 control subjects were enrolled in this pilot study. They were recruited through the University of Utah clinics and surrounding communities via referrals and flyers, the University of Utah study Locator Website, the University of Utah-Center on Aging Participant Registry, and researchmatch.org. The pilot study was approved by the University of Utah Institutional Review Board (#00139658, 05/07/2021) and it was conducted in accordance with the Declaration of Helsinki. Written informed consent was obtained from all participants involved in the study.

Inclusion criteria included age of 18 years and above for all participants. For the depressed sample, a current major depressive episode as identified through the Mini International Neuropsychiatric Interview (MINI) 7.0 (32), and of at least moderate severity based on the Hamilton Depression Rating Scale (HAM-D) (33), was required. Participants were also required to be planning or currently receiving treatment for depression. Presence of psychotic features was not considered an exclusion criterion. We did not differentiate between unipolar and bipolar depression; however, we screened participants for past manic and hypomanic symptomatology. None of our participants met criteria for current or past manic/hypomanic episodes, and only one participant reported past hypomanic symptoms without meeting full criteria. Study exclusion criteria included poorly controlled medical conditions, pregnancy, a diagnosis of schizophrenia or schizoaffective disorder, history of traumatic brain injury or other neurological signs during the past year, MRI contraindications, substance use disorder during the past 3 months, neurocognitive disorder diagnosis during the past year, and diagnosis of autism or intellectual disability. Additionally, specifically to the control group we excluded participants with bipolar disorder, major depressive disorder (lifetime diagnosis), post-traumatic stress disorder (PTSD), obsessive compulsive disorder (OCD), eating disorder (current) and daily use of psychotropic medication.

All participants were screened with the MINI in order to confirm that inclusion/exclusion criteria were met. Individuals with depression underwent a subsequent phone/video conference interview with a psychiatrist or psychiatry resident, during which the 17 and 24 item HAM-D; were administered to validate the severity of depression. After participant screening, but prior to their in-person visit, participants completed the Patient Health Questionnaire-9, a self-reported tool designed to screen for and measure the severity of depression, online (34). Out of the 20 depressed participants, only 17 completed the HAM-D scales due to scheduling conflicts and withdrawing prior to their appointment. Through the MINI, we gathered information on past and recurrent depressive episodes. Of the 20 depressed participants, 18 met criteria for at least one past major episode of depression, and 16 met criteria for recurrent major depressive episodes. On average, depressed participants reported 15.9 depressive episodes over their lifetime. (**Supplementary Table S7**).

### 2.2 Behavioral measures

#### 2.2.1 Gait

The 4 meter walk test (35) was used to measure gait speed. For this test, participants walked over a straight, 8-meter course with markers at 2m, 6m, and 8m that were placed on the floor. Participants started at the 0m end of the course and were instructed to walk 8 meters overall. Walking duration was recorded once the subject’s leading foot crossed over the 2m marker and ended when the subject’s leading foot crossed the 6m marker. The initial two meters (0m - 2m) allowed subjects to reach a steady walking pace before recording. The last two meters (6m - 8m) were included to prevent a premature decline in speed while recording. Participants completed three trials at normal walking pace and three trials at maximum gait speed without running. The average velocity (m/s) across all three trials was calculated as the outcome measure.

We also used the 2-minute walk test to measure distance traveled at a casual gait speed. For this test, participants were asked to walk at their regular walking pace over a straight walking path of 40 feet of which the beginning and the end were marked. Participants were asked to make a 180 degree turn and continue toward the other end each time to reach the end of the path, repeating this cycle once for a duration of two minutes. The outcome measure was total distance traveled in meters.

Finally, we used the walking-while-talking (WWT) test (36) to measure motor-cognitive dual-tasking. The WWT test involved reciting alternate letters of the alphabet. Subjects were requested to walk a 16-meter course with a turn in the middle. The course was set out with markers at 2m and 8m. Subjects were requested to start walking at 0m and at the 2m mark, the timer was started. From there, subjects were instructed to recite every other letter from the alphabet, while continuing to walk to the 8m mark, make a turn, and then walk back over the 2m mark where the timer was stopped. Participants were instructed to pay equal attention to both walking and reciting. They completed three trials that differed in the starting letter of the alphabet, which was A, B, or N for trials 1, 2, and 3, respectively. Performance was calculated based on the total number of letters recited, the number of correct letters, and the ratios of total transitions per second and correct transitions per second. Velocity (m/s) during the WWT task was also calculated as distance covered in each trial divided by walking time for each respective trial. Dual-task cost was determined as the difference in velocity in meters per second between the normal pace walking on the 4-meter walking test and the dual-task. The average measure across the three trials was calculated for all outcomes of the WWT task. Because one depressed subject did not adhere to the task instructions, data for this participant were removed from analyses.

#### 2.2.2 Sarcopenia

Hand grip strength was obtained in pounds using a hydraulic Jamar Digital Hand Dynamometer. Participants were asked to sit comfortably in a standard chair with legs, back support and fixed armrests. They were instructed to rest their forearms on the arms of the chair with their wrist over the edge of the armrest and their thumb facing upwards. During each trial, participants were required to grip the dynamometer as long and as tightly as possible until the number on the display did not increase further (this generally happened after ~5 sec). Participants completed three trials per hand, alternating between them after each recorded trial. All participants began with their left hand. The maximum performance for participants’ dominant and non-dominant hand across the three trials was used as the outcome measure for this analysis.

Knee extension strength was collected in pounds with the Lafayette Instrument Hand-Held Dynamometer (Model 01165). Participants were instructed to lie supine on a padded mat, with a bolster positioned under the knee completing the trial. They had their tested knee with the bolster fixed at an angle of 35° and their untested knee flat on the mat. Before each trial, the dynamometer was placed on the tibia, proximal to the medial malleolus. During recording, participants were informed that once the trial began, they would hear a tone from the device, then a second tone to start extending their knee with as much force as possible until they heard two tones in rapid succession, indicating the end of the trial. All participants started with their left knee and alternated between left and right for six trials in total. The amount of maximum force generated during each trial was recorded in pounds. The maximum performance of participants’ dominant and non-dominant leg across the three trials was used as the outcome measure.

#### 2.2.3 Fine motor skills

The 9 Hole Peg Test was administered to record dexterity and fine motor skills by recording the time for subjects to place and remove nine pegs within nine holes. For this task, the JAMAR pegboard was used during data collection. This consisted of a design with a 3x3 grid of holes with a receptacle holding nine pegs adjacent to it. Participants were instructed to place their hand next to the board and to move each peg, one at a time to each hole once the trial began. Once all pegs were placed, the participant was required to remove each peg one at a time back to the receptacle. Participants were allowed to place and remove the pegs in any order of their choosing. Participants began by using their dominant hand and were given one practice trial before the recorded time trial began. Six-time trials were then completed, alternating between dominant and non-dominant hands. The time taken by each participant to place and remove each peg was recorded in seconds and used as an outcome measure.

An in-house developed computerized Archimedes Spiral Test was conducted to measure speed and accuracy functions of fine motor skills. The testing materials consisted of a spiral template that was printed on a piece of paper attached to an electronic drawing board (WACOM Graphire Wireless Pen Tablet, model CTE630BT). Participants were instructed to place the pen in the middle of the spiral before the tracing started. They were not allowed to lean on the drawing board with their hand or arm and were asked to trace the spiral as accurately and as fast as possible over three trials using their dominant hand. Automatic quantitative analyses were performed using custom-made software written in MATLAB (version 8.1; The Mathworks, Natick, MA, USA) (37–39). This yielded the following outcome measures: movement time (s), defined by the time it took the participant to trace the spiral; length of drawing (cm), defined as the length of the drawn spiral; average speed, defined by the ratio of length of drawing and movement time; speed variability (cm/s), defined as the standard deviation of the instantaneous speed; deviation from template (in pixels), defined as the area between the template and the drawn spiral; number of crossings, defined as the number of times the drawn spiral crossed the template; and return movements, defined as the absence or presence of one or more return movements (i.e., brief movements in the opposite direction). For the analysis, the minimum value across the three trials was calculated for movement time, return movements, and the number of times participants crossed the template. Similarly, for the deviation from the template outcome, the minimum value of the average, sum, and standard deviation of the absolute radial distance between the template and the spiral (in pixels) across the trials was used. The maximum value of the average speed across the three trials was also calculated for the analysis.

#### 2.2.4 Cognitive function

The Symbol Digit Modalities Test (SDMT) (40) was used to measure processing speed. In this symbol/number coding task, participants are presented with a key containing nine unique geometric shapes paired with numbers ranging from 1-9. They are instructed to use this key to match symbols that are presented in a sequence to their corresponding numbers, without skipping any items. After completing a practice run of 10 items, participants have 90 seconds to complete as many items in consecutive order as possible. The outcome measure for the SDMT is the total number of correctly completed items.

We used the Hopkins Verbal Learning Test Revised (HVLT-R) (41, 42) to test short- and long- term verbal memory and learning. Across three trials, participants were presented with a list of 12 words that were read to them and instructed to verbally recall as many words as they could. Without prior notification, after 25 minutes elapsed, participants were asked to report how many words they can remember to test delayed recall. The outcome measures for the HVLT-R were age adjusted T-scores (scaled scores) for the total number of immediate and delayed correctly recalled words. Scaled scores were derived from the HVLT-R professional manual.

The Brief Visuospatial Memory Test Revised (BVMT-R) (43) was used to test visual memory and learning. For this task, participants were asked to carefully study a sheet with six figures. They have 10 seconds and then are asked to draw each figure exactly as it appeared (shape) and place it in the same area as it was presented on the sheet (location). This was repeated for three trials. Without prior notification, after 25 minutes elapsed, participants were asked to try to draw as many figures as they could remember in the correct location and shape. The outcome measures for the BVMT-R were age adjusted T-scores (scaled scores) for the total number of immediate and delayed correctly drawn designs. Scaled scores were derived from the BVMT-R professional manual.

Finally, we used the D-KEFS Color-Word Interference Test (44) to measure cognitive interference. This task consists of four trials, beginning with the color naming trial, during which participants are presented with a page containing a series of red, green, and blue squares. Participants are asked to say the names of the colors as quickly as they can without making mistakes. This is followed by the word reading trial, where they are presented with a page containing the words “red,” “green,” and “blue” printed in black ink and are asked to read the words aloud as quickly as they can without making mistakes. Subsequently, participants complete the inhibition trial, where they are shown a page containing the words “red,” “green,” and “blue” printed incongruently in red, green, or blue ink. They are asked to say the color of the ink in which each word is printed as quickly as they can without making mistakes. Last is the inhibition/switching trial, where a page containing the words “red,” “green,” and “blue” written in red, green, or blue ink is provided to participants. Half of these words are enclosed within boxes, while the other half are presented without. Participants are asked to say the color of the ink in which each word is printed (if unboxed), but to read the word aloud (and not name the ink color) when a word appears inside a box, as quickly as they can without making mistakes. As outcome measures, we used the time to complete each different trial and the number of self-corrected and uncorrected responses at each trial. Each timed trial results were converted to scaled scores based on participants’ age and trial duration, following the normative tables provided within the D-KEFS Examiner manual.

### 2.3 Hand dominance

Hand dominance was determined using the Dutch handedness questionnaire (45). This is a 16-item self-assessment handedness questionnaire asking which hand is being used for certain operations (e.g., opening a jar). Scores of -1, 0, and 1 were assigned to items for which the subject selected left hand, ambidextrous, and right hand, respectively. Final scores were binarized (left-handed vs. right-handed) by averaging the scores on the 16 items and selecting left-handedness for a negative mean score and right-handedness for a positive mean score.

### 2.4 Procedure

Once participants were determined eligible for participation, electronic informed consent was completed. On the day of their visit, participants completed the following behavioral assessments: 2-minute walk test, 4-meter walk test, WWT test, SDMT, D-KEFS Color-Word Interference Test, 9-Hole Peg Test, HVLT-R, Archimedes Spiral Test, BVMT-R, grip strength, and knee extension strength. Subjects received $90 compensation for completing the entire study.

### 2.5 Statistical analysis

Statistical analysis was performed in R version 4.2.2 (2022-10-31). Prior to statistical analysis, all data were evaluated for outliers, but this did not result in any exclusions. Group differences in potentially confounding continuous and categorical variables were compared using t-tests and chi-square tests, respectively. Both the t-tests and chi-square tests were conducted using functions from base R (R core tools). Group differences in behavioral measures were tested using linear regression models adjusted for age, sex, and education. The η^2^ value for the group factor, i.e., the proportion of variance in the model explained by group, is reported as a measure of effect size for the group comparisons. Association between depression severity and behavioral measures was tested using linear regression models, also adjusted for age, sex, education. For these regression models we used behavioral measures as the outcome and depression severity (HAMD-24 scores) as the predictor. The η^2^ value for the depression severity, i.e., the proportion of variance in the model explained by the HAMD-24 score, is reported as a measure of effect size for the HAMD-24 score. An alpha threshold of 0.05 (two-tailed) was used for all hypotheses. Models were adjusted for multiple comparisons using false discovery rate (FDR) correction (46).

## 3 Results

### 3.1 Demographic and clinical characteristics

An overview of the demographic information is presented in **Table 1**. There were no significant differences in mean age, years of education completed, nor sex distribution differences between the control and depressed participants. Participants in the depression group showed significantly more symptoms of depression on the PHQ-9 than controls. For three individuals from the depression group, the HAM-D interviews were not completed due to scheduling conflicts and withdrawing prior to their appointment.

**Table 1:** Demographic and clinical characteristics of the present sample.

Variable	Metric	Controls (n=22)	Depressed (n=20)	p-value	Cohen’s d	Total sample
Age (19.2-58.3)	m(sd)	31.8 (10.2)	30.6 (11.1)	0.71	0.11	42
Sex (M/F)	N	7/15	8/12	0.82	0.18	15/27
Education	m(sd)	17.1 (1.8)	15.8 (3.2)	0.1	0.54	42
PHQ-9	ms(sd)	2.5 (2.3)	15.8 (5.9)	0<0.001	3.04	42
GAD-7	ms(sd)	2.5 (3.7)	11.5 (5.1)	0<0.001	2.02	42
HAM-D 17	m(sd)	–	16.64 (4.9)	–	–	17
HAM-D 24	m(sd)	–	24.1 (6.7)	–	–	17

### 3.2 Group differences in cognitive and motor behavioral outcome measures

#### 3.2.1 Cognition

Group differences in behavioral outcome measures in cognitive domains are displayed in **Table 2**. Significant differences in performance were observed in the color naming and cognitive interference conditions of the D-KEFS Color-Word Interference test, as well as in the delayed recall of the HVLT-R. In all above instances, the depressed participants performed worse than the controls; however, these results did not survive FDR correction. No significant differences were observed in the remaining cognitive function measures.

**Table 2:** Group differences in cognitive performance.

Variable	Depressed mean (sd)	Control mean (sd)	Beta value	p-value	η^2^
Symbol Digit Modalities Test (# correct)	54.26 (12.01)	58.82 (6.31)	-4.393	0.151	0.049
D-KEFS Color-Word Interference Test (scaled score)					
Color naming	8.85 (3.03)	10.68 (2.78)	-2.196	**0.024**	0.125
Word naming	9.70 (2.70)	11.09 (2.04)	-1.365	0.087	0.073
Cognitive interference	9.60 (2.72)	11.32 (2.10)	-1.947	**0.016**	0.139
Inhibition/Switching	9.35 (2.68)	11.05 (2.40)	-1.482	0.081	0.074
Hopkins Verbal Learning Test - Revised (Age-Corrected T-score)					
Total recall	41.85 (12.19)	46.77 (9.58)	-3.454	0.296	0.028
Delayed recall	41.89 (13.86)	50.05 (11.02)	-6.973	**0.016**	0.144
Brief Visuospatial Memory Test - Revised (Age-Corrected T-score)					
Total recall	51.50 (11.70)	55.68 (8.19)	-4.682	0.200	0.042
Delayed recall	51.60 (10.71)	58.48 (5.23)	-8.197	0.054	0.094

#### 3.2.2 Gait

Group differences in the gait domain outcome measures are displayed in **Table 3**. Results showed no significant differences between depressed participants and controls for any of the gait measures.

**Table 3:** Group differences in the gait domain.

Variable	Depressed mean (sd)	Control mean (sd)	Beta value	p-value	η^2^
Two-minute Walk Test	126.38 (18.20)	125.93 (17.58)	-1.120	0.848	0.001
4 Meter Walk Test					
Maximum speed (m/sec)	1.87 (0.26)	1.96 (0.38)	-0.064	0.550	0.009
Normal pace (sec)	2.88 (0.56)	2.80 (0.64)	0.092	0.636	0.006
Walking While Talking Test (WWT)					
Dual-task cost	0.48 (0.37)	0.54 (0.34)	-0.040	0.716	0.003
Correct transition trial avg	6.37 (1.94)	6.09 (1.58)	0.563	0.318	0.025
Correct transition ratio avg	0.48 (0.11)	0.48 (0.13)	0.012	0.754	0.002
Total transition trial avg	8.17 (1.77)	8.48 (1.78)	-0.025	0.965	0.000
Total transition ratio avg	0.62 (0.11)	0.68 (0.14)	-0.045	0.284	0.029
Dual-task walk speed avg	0.97 (0.36)	0.97 (0.18)	-0.019	0.842	0.001

#### 3.2.3 Sarcopenia

Group differences in the sarcopenia domain outcome measures are displayed in **Table 4**. There were no significant differences observed between depressed participants and controls in the sarcopenia outcome measures.

**Table 4:** Group differences in the sarcopenia domain.

Variable	Depressed mean (sd)	Control mean (sd)	Beta value	p-value	η^2^
Grip Force Maximum Performance					
Dominant hand	74.75 (21.08)	71.92 (19.16)	2.214	0.683	0.003
Non-dominant hand	70.70 (20.60)	58.85 (30.96)	11.991	0.112	0.045
Knee Extension Maximum Performance					
Dominant leg	59.51 (17.55)	64.69 (19.88)	-6.206	0.289	0.026
Non-dominant leg	60.05 (16.36)	64.58 (19.43)	-5.818	0.322	0.025

#### 3.2.4 Fine motor function

Group differences in the fine motor function domain outcome measures are displayed in **Table 5**. Compared to control participants, those with depression were significantly slower at the 9 Hole Peg Test both for the dominant and nondominant hand. These differences survived FDR correction for the nondominant hand. No group differences in Archimedes spiral tracing performance were observed.

**Table 5:** Group differences in the fine motor function domain.

Variable	Depressed mean (sd)	Control mean (sd)	Beta value	p-value	η^2^
9 Hole Peg Test (Minimum Performance)					
Dominant hand	18.32 (1.84)	17.12 (1.78)	1.153	**0.044**	0.088
Non-dominant hand	21.66 (2.74)	19.01 (2.03)	2.961	**0.001**	0.227
Archimedes Spiral Tracing Test					
Average velocity, pixels/second	587.53 (249.18)	416.14 (216.53)	159.400	0.055	0.099
Total movement time (sec)	5.76 (3.93)	8.53 (4.76)	-2.970	0.053	0.098
# movements of opposite direction	0.06 (0.24)	0.05 (0.21)	0.034	0.665	0.005
# times the spiral crosses the template	2.71 (1.45)	3.32 (1.25)	-0.529	0.257	0.035
Average absolute radial distance between template and spiral (pixels)	7.29 (3.44)	6.36 (1.99)	1.124	0.240	0.040
Sum of absolute radial distance between template and spiral (pixels)	3721.59 (1107.33)	4912.00 (2284.45)	-972.823	0.123	0.059
SD of absolute radial distance between template and spiral (pixels)	5.35 (3.22)	4.32 (1.43)	1.110	0.183	0.050

Bold values are statistically significant (p <0.05).

### 3.3 Association between depression severity and behavioral measures

**Figure 1:**
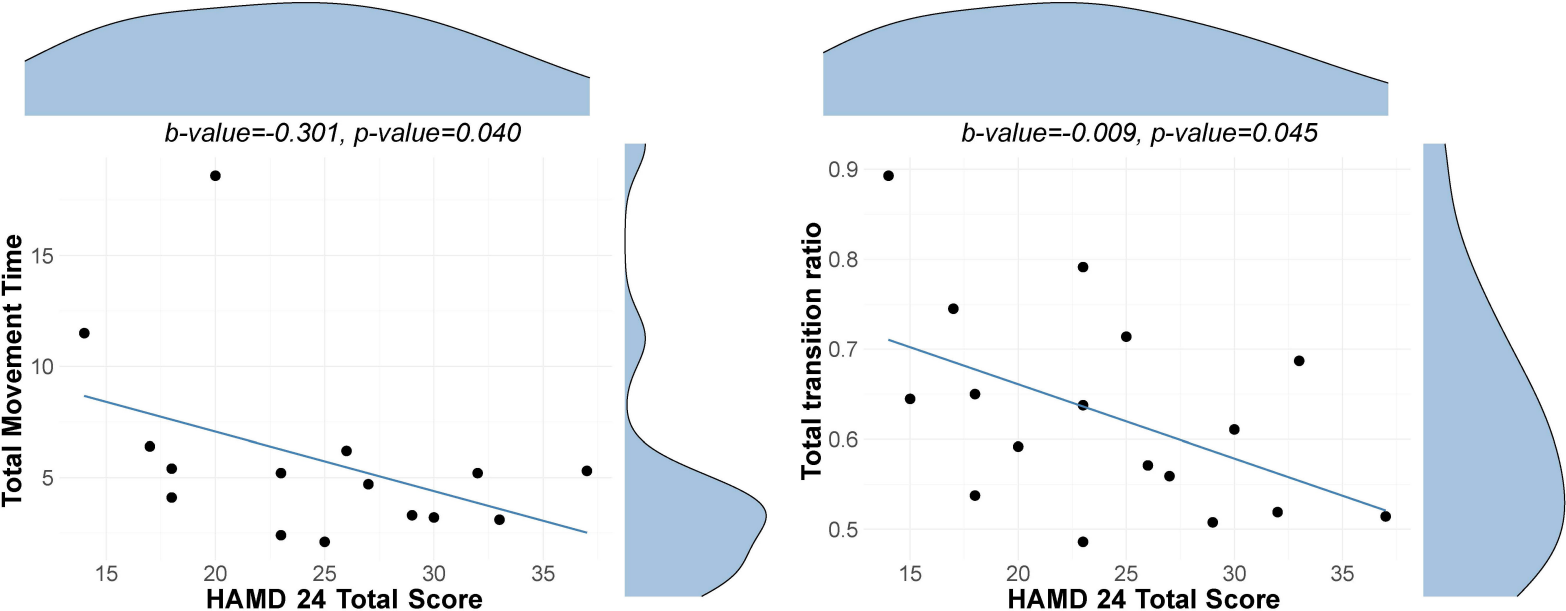
Association between depression severity and behavioral measures. Scatterplots with distribution density curves for the HAMD-24 total score (top) and behavioral measure (right-hand side). Left Scatter plot with a regression line showing the association between depression severity as indicated by HAM-D 24 scores and total movement time in the Archimedes Spiral Test. Right Scatter plot with a regression line showing the association between depression severity and total transitions ratio in the walking while talking test. Note that the movement time reflects the minimum movement time across the three trials, and the ratio reflects the average across three trials.

Significant associations between depression severity and behavioral performance within the depressed sample are displayed in **Figure 1**. Greater severity of depression is associated with poorer performance on the WWT dual cognitive-motor task within the gait domain and quicker movement times in the Archimedes Spiral task within the fine motor function domain; however, these associations did not survive FDR correction. No significant associations were observed for the remaining behavioral measures. Detailed results are shown in **Supplementary Tables S1**-**S4**.

## 4 Discussion

This pilot study aimed to examine how motor and cognitive abilities are affected in depression. To this end, a group of depressed individuals with and a group of healthy controls performed several objective motor and cognitive tests. The present results show that, in comparison to controls, depressed participants performed worse on measures of executive function, verbal memory, and fine motor abilities, but not in terms of gait or sarcopenia. Within the depressed group, it was observed that greater depression severity was associated with poorer cognitive-motor dual-tasking performance and faster movement time on the Archimedes spiral task assessing fine motor function.

### 4.1 Depressed participants show significant fine motor impairments, but no deficits in other motor domains

Depressed participants performed worse on the 9 Hole Peg Test reflecting poorer fine motor abilities. Fine motor alterations have been established as a feature of depressive symptomatology (47), yet few studies have assessed them using objective, standardized measures. Fine motor problems can substantially affect daily functioning, impacting routine tasks, work productivity, and social and recreational activities (48). Although a clear relationship between psychomotor slowing and poor functional outcomes has been established in schizophrenia, this connection has not been specifically investigated in depression. Studies that examine specific domains of motor function, such as fine motor skills, and the degree of functional disability they account for could help us better understand the contribution of motor function to the disease burden of depression. Furthermore, fine motor alterations may also interfere with specific treatments for depression. For example, an evidence-based treatment called behavioral activation encourages individuals to engage more consistently in enjoyable activities and fulfill meaningful life roles. This approach aims to disrupt patterns of avoidance, withdrawal, and inactivity that are common in depression (49). However, individuals with fine motor disturbances may find it challenging to participate in certain activities (e.g., drawing, playing video games), thus potentially hindering the effectiveness of behavioral activation treatment. Moreover, theories on social functioning in depression suggest that a lack of skills in a particular domain may lead to low self-acceptance (50) and potentially interact negatively with emotional processing (51). The ways in which motor disturbances interact with other domains affected in depression—such as positive valence, social functioning, and arousal—remain largely unexplored (52). However, investigating these relationships could deepen our understanding of the role of motor function in depression and inform the development of targeted treatments to address these specific impairments.

There are several potential explanations for the lack of a statistical group difference in gait and sarcopenia motor domains. First, previous literature reports that increased gait variability during dual-tasking has been demonstrated in older depressed patients (20, 36) with depression also being an independent risk factor for falls in elderly patients. This suggests that motor brain reserves are probably affected more significantly later in life (67.2 ± 3.6 years) (53). Because with a mean age of 32.8 years, our sample was much younger on average these motor deficits may not have fully emerged yet. Another explanation could be that the motor task was too simple. Walking is considered to be a relatively automatic process (54). This is supported by the fact that during earlier developmental stages (childhood), where this automatization is under development, more profound variability in gait measures has been found (55, 56). Interestingly, a study examining the motor characteristics of various phases of bipolar disorder found significantly faster gait during the hypomanic phase. However, no significant differences were observed during the depressed phase. The authors suggest that both psychomotor agitation and retardation may co-occur during depression, potentially exerting opposing effects (57). Additionally, sarcopenia has been linked to depression, primarily because depressive symptoms such as fatigue, poor appetite, and low motivation can result in decreased physical activity and increased periods of sitting or lying down. Over time, these behaviors may lead to sarcopenia (58). However, our study found no significant differences in this domain, which may be explained by the characteristics of our sample. Since our participants were community-dwelling patients and not hospitalized, their depression may not have been severe enough for the effects leading to sarcopenia to become apparent.

### 4.2 Depressed participants exhibit significant decrements in processing speed, cognitive interference, and memory

It was also observed that depressed participants exhibited poorer performance in the color-naming and cognitive interference (inhibition) trials of the D-KEFS Color-Word Interference task, but their performance was not significantly different in the word-naming and inhibition/switching trials. It is widely accepted that color naming is more time-consuming than word naming, as it is thought to involve more complex cognitive processes (59). Moreover, although the inhibition/switching trial is generally considered more challenging, there is anecdotal evidence suggesting otherwise. The inhibition trial, which heavily involves color naming and is more time-consuming, precedes the inhibition/switching trial. Here, participants might have already gained practice with the inhibition process. These findings suggest that the cognitive interference (inhibition) trial also requires more cognitive resources (60). This shows that under conditions of greater cognitive demand, participants with depression underperformed compared to controls. Our results of reduced processing speed and cognitive interference in depression corroborate the existing literature on neurocognitive impairment in depression. Prior studies indicate that cognitive difficulties not only contribute to psychosocial deficits, but that they also decrease the probability of remission and recovery from a depressive episode (25, 61). Moreover, a recent study using a letter sorting test with different levels of difficulty showed that increased cognitive effort was associated with lower momentary mood (62). Collectively, these findings underscore how cognitive impairments compound the functional impairments caused by motor deficits in depression. Studies on other disorders with psychomotor changes, such as Parkinson’s disease and Huntington’s disease (63, 64), which often co-occur with depression, suggest that psychomotor disturbances may reflect specific biological underpinnings involving neural circuits like the basal ganglia. A recent study demonstrated that individuals with depression show distinct connectivity patterns within the motor network, even in remission (65). These findings indicate that both cognitive and motor dysfunctions persist in remitting states, underscoring the importance of developing interventions targeting both cognitive and motor functions in depression.

Notably, depressed participants exhibited poorer performance on the delayed recall portion of the HVLT task. While verbal memory deficits are typically associated with late-life onset of depression (66), findings in younger patients have been less consistent. However, such decrements in late-life depression have been linked to an increased risk of developing Alzheimer’s disease (67, 68). Given that the mean age of our sample is 32.8 years, our results suggest that memory difficulties can also manifest in younger individuals with depression. This highlights the importance of early interventions in the treatment of depression to potentially mitigate these cognitive impairments.

### 4.3 Severity of depression predicts poorer dual-task performance but unexpected patterns in spiral drawing performance

Within the depressed sample, we observed that greater severity of depression correlated with poorer performance on the WWT dual cognitive-motor task within the gait domain and quicker movement times in the Archimedes Spiral task within the fine motor function domain. More specifically, higher depression severity was linked to fewer transitions per second in the WWT aspect of the dual-task. This part of the task predominantly measures the cognitive component of movement (20), indicating that decrements in this area may reflect not only restricted cognitive resources but also a diminished capacity to allocate these resources effectively during multitasking (69, 70). These findings suggest diminished cognitive control in individuals with more severe depression and support the idea that psychomotor difficulties reflect a more severe depression phenotype. Additionally, this could also reflect the relationship between cognitive and motor functions, potentially demonstrating how cognitive decrements underlie motor ones in depression. Interestingly, despite significant associations with the cognitive component of the dual task, we did not observe a significant relationship between depression severity and dual-task cost, calculated as the difference in walking speed between the 4-meter walk and the dual-task condition. A more nuanced method proposed by Brauner et al., the P-index (71), weighs dual task performance by cognitive accuracy and may thus offer a more sensitive metric. This promising approach could be applied in future studies.

Interestingly, in contrast to our expectations, faster movement times on the Archimedes Spiral task were associated with greater severity of depression. This finding could have several possible explanations. One potential factor is poor motivation and effort, which are often observed in depressed individuals and could interfere with their ability to fully engage in the task (72). This phenomenon suggests that the apparent speed in task completion may not necessarily reflect enhanced motor function, but rather a reduced quality of task engagement driven by the underlying symptoms of depression. This could also explain why no group differences in performance on the Archimedes spiral test were observed, as the group of depressed participants comprises both mildly and moderately severely depressed.

### 4.4 The need for precision in defining psychomotor disturbances in psychiatric research

Taken together, our findings underscore the need to clarify what constitutes psychomotor disturbance in depression. Although psychomotor symptoms are frequently referenced in both clinical and research settings, the term itself remains poorly defined and is often used to describe a wide range of motor and cognitive symptoms without clear distinction (18, 73). Some researchers argue that the term should focus only on the motor aspects of psychomotor retardation. However, others believe this view is too narrow, as cognitive factors are closely linked to motor symptoms (74). Moreover, psychomotor symptoms are often intertwined with other aspects of depression, such as fatigue, motivation, and impaired attentional processes, with no clear boundaries (75, 76). For example, some clinical measures of psychomotor disturbances evaluate the cognitive aspect, including attentional tests [e.g. Trail Making Test (77), Posner’s covert orientation of visual attention test (COVA) (78)], while others do not [e.g. Nufferno Speed test (79)] (80, 81). By using objective measures of cognitive and motor domains, this study contributes to the process of disentangling these overlapping functions. Future studies should also include measures that assess cognitive processes underlying motor functioning, to further elucidate psychomotor functioning in depression.

### 4.5 Strengths and limitations

A notable strength of our study is that we used a series of objective cognitive and motor behavioral tasks, spanning multiple domains. While prior studies have investigated different cognitive domains within a single depressed sample [e.g., (82–84)], and some studies report on multiple motor functions in depression (85, 86), to our knowledge this study is the first to investigate different motor domains in a depressed adult sample using objective behavioral tasks. Moreover, the tasks utilized are reflective of many daily activities and daily functioning and can directly serve as a functional outcome measure (87).

There are certain limitations to this study. Cognitive and motor function was studied in participants with a currently active depressive episode. An open question therefore remains whether our findings generalize to other depression states (e.g., remitted). Previous studies indicated that cognitive deficits in depression are long-lasting and could persist even if patients return to the euthymic state. We recommend that future studies examine cognitive and motor outcomes in patients with depression in remission or use longitudinal designs to further investigate whether cognitive and motor outcomes in depression change as the depressive symptoms improve.

Similarly, our current sample size did not allow us to differentiate between potential subtypes of depressive symptomatology. It has been well-established that psychomotor changes are present in a depressive episode, yet not all patients with depression will experience those symptoms. Additionally, psychomotor changes can encompass both agitation and retardation, which have opposite effects. Therefore, distinguishing between depressed patients with psychomotor changes versus those without would also be crucial. We acknowledge that the sample size in this study is small; however, this was a pilot investigation specifically designed to explore preliminary signals of cognitive and motor dysfunction in depression. We believe these early findings provide valuable insights that support the need for further, larger-scale research in this area.

Another limitation of this work is that we did not examine premorbid functioning of the depressed participants. Education level (**Supplementary Table S8**) was included as a covariate in our analysis; however, we cannot rule out that some of the observed performance differences may be explained by other factors unrelated to depression. However, we cannot rule out the possibility that some observed performance differences may be influenced by factors unrelated to depression. Additional sociodemographic variables, such as income and occupation, could provide further insight into premorbid functioning and may prove valuable for future research. An additional confounding factor may be the presence of moderate anxiety in the depressed group, which was not observed in the control group. Given that anxiety can contribute to motor alterations (15), future studies should control for its potential effects. Another significant contributing factor could have been medication effects (88). Studies with longitudinal designs where cognitive and motor performance is measured prior to and during an active depressive episode could resolve this issue. A list of medications used by the depressed and control groups can be found in **Supplementary Tables S5** and **S6**.

A final suggestion for future research relates to better understanding the impact of cognitive and motor decrements on functioning in daily life. While neurocognitive decrements have been documented more extensively in older patients (36, 89) our study and growing evidence suggest that this may start earlier in life (90, 91) when individuals are still actively involved in the workforce community, may have a young family and other social obligations. Therefore, understanding the progression of cognitive and motor function across the lifespan in patients with depression can help us further determine the neurocognitive and motor impact of depression. As suggested by others, including functional outcome measures in different domains [e.g., physical health, psychological health, social relationships, and environment as measured via the WHOQOL (92)] could be very meaningful to this end.

## 5 Conclusions

Taken together, the current study indicates that depressed participants experience cognitive and motor decrements, specifically in processing speed, cognitive inhibition, delayed recall, and fine motor function, but not in episodic memory or gross motor function. These findings align with indications that depression significantly impacts executive functioning and memory and demonstrate a decline in fine motor function. These results are directly related to overall daily functioning and quality of life. Therefore, the current findings highlight the need for more specific interventions targeting cognitive and motor dysfunctions in depression that can improve overall quality of life. Future longitudinal studies are essential to track these disturbances over time and across different stages of symptom improvement, while also assessing potential differences in cognitive and motor outcomes among different psychomotor subtypes of depression.

## Data Availability

The raw data supporting the conclusions of this article will be made available by the authors, without undue reservation.
