# Supplementary material for: Cognitive and motor disturbances in depression: insights from comprehensive behavioral assessments
Source: Front Psychiatry. 2025 Jul 29;16:1624776. doi: 10.3389/fpsyt.2025.1624776 (PMC12340722; doi:10.3389/fpsyt.2025.1624776)
Supplement: Supplementary file 1 [file Table1.docx]

Supplementary Material

# Supplementary Tables

**Table S1**. Association between depression severity and the cognitive domain measures for participants within the MDD group. *No significant associations were found between depression severity and the cognitive domain measures for participants within the MDD group, according to linear regression analysis.*

| **Variable** | **Beta value** | **p-value** | **η^2^** |
| --- | --- | --- | --- |
| **Symbol Digit Modalities Test (# correct)** | -0.342 | 0.512 | 0.032 |
| **Stroop Color Word Test (scaled score)** | | | |
| Color naming | 0.116 | 0.268 | 0.091 |
| Word naming | -0.043 | 0.698 | 0.011 |
| Cognitive interference | 0.062 | 0.564 | 0.025 |
| Inhibition/Switching | 0.037 | 0.693 | 0.009 |
| **Hopkins Verbal Learning Test - Revised (Age-Corrected T-score)** | | | |
| Total recall | 0.733 | 0.125 | 0.161 |
| Delayed recall | 0.574 | 0.154 | 0.148 |
| **Brief Visuospatial Memory Test - Revised (Age-Corrected T-score)** | | | |
| Total recall | 0.036 | 0.940 | 0.000 |
| Delayed recall | 0.180 | 0.747 | 0.009 |

**Table S2.** Depressed Group correlation of depression severity with Gait domain measures*. Greater severity of depression is associated with lower total transition ratio of the WWT dual cognitive-motor task within the gait domain. This result didn’t survive FDR correction. No significant correlations were found for the remaining tests of the gait domain.*

| **Variable** | **Beta value** | **p-value** | **η^2^** |
| --- | --- | --- | --- |
| **Two-minute Walk Test** | -0.725 | 0.333 | 0.069 |
| **4 Meter Walk Test** | | | |
| Maximum speed (m/sec) | 0.003 | 0.769 | 0.007 |
| Normal pace (sec) | 0.025 | 0.304 | 0.085 |
| **Walking While Talking Test (WWT)** | | | |
| Dual task cost | 0.013 | 0.449 | 0.047 |
| Correct transition trial avg | 0.071 | 0.376 | 0.050 |
| Correct transition ratio avg | -0.003 | 0.558 | 0.025 |
| Total transition trial avg | 0.056 | 0.357 | 0.046 |
| Total transition ratio avg | -0.009 | **0.045** | 0.264 |
| Dual task walk speed avg | -0.027 | 0.084 | 0.214 |

**Table S3.** Depressed Group correlation of depression severity with Sarcopenia domain measures. *No significant correlations were found between depression severity and the sarcopenia domain measures for participants within the MDD group.*

| **Variable** | **Beta value** | **p-value** | **η^2^** |
| --- | --- | --- | --- |
| **Grip Force Maximum Performance** | | | |
| Dominant hand | 0.960 | 0.137 | 0.080 |
| Non-dominant hand | 0.939 | 0.112 | 0.083 |
| **Knee Extension Maximum Performance** | | | |
| Dominant leg | 0.518 | 0.426 | 0.031 |
| Non-dominant leg | 0.515 | 0.413 | 0.036 |

**Table S4.** Depressed Group correlation of depression severity with Fine motor domain measures. *Greater severity of depression is associated with quicker movement times in the Archimedes Spiral task within the fine motor function domain. This result didn’t survive FDR correction. No significant correlations were found for the remaining tests of the fine motor function domain.*

| **Variable** | **Beta value** | **p-value** | **η^2^** |
| --- | --- | --- | --- |
| **9 Hole Peg Test (Minimum Performance)** | | | |
| Dominant hand | 0.001 | 0.988 | 0.000 |
| Non-dominant hand | 0.071 | 0.458 | 0.037 |
| **Archimedes Spiral Tracing Test** | | | |
| Average velocity, pixels/second | 14.957 | 0.169 | 0.140 |
| Total movement time (sec) | -0.301 | **0.040** | 0.219 |
| # movements of opposite direction | 0.012 | 0.300 | 0.087 |
| # times the spiral crosses the template | -0.008 | 0.896 | 0.001 |
| Average absolute radial distance between template and spiral (pixels) | 0.136 | 0.291 | 0.060 |
| Sum of absolute radial distance between template and spiral (pixels) | -68.389 | 0.151 | 0.151 |
| SD of absolute radial distance between template and spiral (pixels) | 0.108 | 0.352 | 0.043 |
| Average velocity, pixels/second | 14.957 | 0.169 | 0.140 |

**Table S5.** List of medications reported by the depressed participants.

| **Participants** | | **Medications** |
| --- | --- | --- |
| 1 | Sertraline, Metformin | |
| 2 | Losartan, Atorvastatin, Brexpiprazole, Bupropion, Venlafaxine, Lamotrigine, Apixaban | |
| 3 | Sertraline, Levothyroxine | |
| 4 | Amphetamine/dextroamphetamine mixed salts, Venlafaxine, Buspirone, Lithium | |
| 5 | Sertraline, Lamotrigine, Irbesartan-Hydrochlorothiazide | |
| 6 | Escitalopram, Bupropion, Lithium, Loratadine, Alprazolam, Emtricitabine/tenofovir | |
| 7 | Escitalopram | |
| 8 | Bupropion, Allopurinol, Meloxicam, Propranolol, Buprenorphine | |
| 9 | Amphetamine/dextroamphetamine mixed salts, Bupropion | |
| 10 | Lisdexamfetamine, Escitalopram, Trazodone, Metoprolol | |
| 11 | Escitalopram | |
| 12 | Adalimumab, Duloxetine, Zolpidem | |
| 13 | Sertraline | |
| 14 | Tizanidine, Bupropion, Buspirone, Trazodone | |
| 15 | Amphetamine/dextroamphetamine mixed salts | |
| 16 | Duloxetine | |

**Table S6**. List of medications reported by control participants.

| **Participants** | **Medications** |
| --- | --- |
| 1 | Albuterol, Budesonide/formoterol |
| 2 | Spironolactone |
| 3 | Bictegravir/emtricitabine/tenofovir alafenamide |
| 4 | Amphetamine, dextroamphetamine mixed salts |

**Table S7. Prevalence of diagnostics and symptoms among depressed participants.**

|  | **Current major depressive episode** | **Past major depressive episode** | **Recurrent major depressive episode** | **Current Suicidal ideation/MINI** | **Current Suicidal ideation/HAMD** | **Current antidepressant medication** |
| --- | --- | --- | --- | --- | --- | --- |
| **N (present/total)** | 20/20 | 18/20 | 16/20 | 12/20 | 12/17 | 15/20 |
| **Percent %** | 100 | 90 | 80 | 60 | 70.6 | 75 |

**Table S8. Education level of depressed and control participants.**

| **Group** | **Some College, no degree** | **Associate’s degree** | **Bachelor’s degree** | **Master’s degree** | **Professional Doctorate degree** | **Academic Doctorate degree** |
| --- | --- | --- | --- | --- | --- | --- |
| **Control** | 3 | 1 | 9 | 7 | 1 | 1 |
| **Depressed** | 5 | 3 | 10 | 0 | 1 | 1 |
